# The Treatment of Gastric Ulcer

**Published:** 1893-02-25

**Authors:** 


### THE HOSPITAL CLINIC.

[*The Editor will be glad to receive offers of co-operation and contributions from members of the profession. All letters should be addressed to* THE EDITOR, THE LODGE, PORCHESTER SQUARE, LONDON, W.]

### ST. MARY'S HOSPITAL.

### THE TREATMENT OF GASTRIC ULCER.

The treatment of gastric ulcer as conducted at St. Mary's Hospital will, for the sake of convenience and clearness, be considered under the following arrangement: 1. The hygienic and dietetic treatment. 2. The medicinal treatment. 3. The treatment of the complications.

*The Hygienic and Dietetic Treatment.*—It is an essential point in the treatment of gastric ulcer that the patient be rigidly confined to bed for some weeks. This must be strictly enforced in all cases in which gastric ulcer is suspected, even in the absence of hæmatemesis or severe vomiting. After three or four weeks the patient may be allowed to sit up for a few hours every day. Exercise must be resumed very gradually, and exertion of any sort must be carefully avoided during the early stages of convalescence.

At St. Mary's it is the custom with respect to diet to treat each case on its merits. In those cases in which pain and vomiting are severe, it is usual to withhold all food by the mouth, and to feed the patient by nutrient enemata. The length of time during which this treatment can be carried out varies according to the severity of the symptoms, and the strength of the patient; but, as a rule, it is not found necessary to continue it for more than a week or ten days. The enema most commonly used is one consisting of a mixture of 2 oz. to 3 oz. of strong beef-tea, two raw eggs, and some brandy (℥j), to which may be added 2 oz. of peptonised milk. The enema should not exceed six ounces. It may be administered every six hours, and the rectum must be washed out by means of a simple enema once in the twenty-four hours. More satisfactory results are obtained from the use of these enemata, than from nutrient suppositories alone, though the latter may sometimes be given with advantage alternately with the former. Thirst, which is sometimes considerable, may be assuaged by sucking small pieces of ice, or the patient may be allowed to drink sparingly of toast or barley water. After the acute symptoms have subsided an attempt may be made to give food by the mouth. This at first should consist of milk, diluted with lime or barley water, or it may be given peptonised. The food must be given in small quantities (2-3 oz.) at a time, and at frequent and regular intervals. At the outset it may be given every two hours, the interval being gradually increased as convalescence is established. This treatment must be contined for at least two or three weeks, after which the dietary may be cautiously increased, both in quality and quantity. Egg may be given at first, lightly boiled or beaten up with the milk, or in the form of custard pudding, Cocoa, boiled fish, or boiled chicken may be gradually added. Tea, green vegetables, cheese, spices, &c., are better entirely avoided for some months. Alcoholic liquors of all kinds must be prohibited, so long as the patient suffers from dyspeptic symptoms.

*The Medicinal Treatment.*—Pain may be relieved by the application of hot poultices or turpentine stupes to the epigastrium. Should these measures fail, the application of a blister to the same region may be employed.

It is usual at St. Mary's to commence by giving a mixture containing bismuth (gr. x.-xx.) and sodæ bicarb. (gr. xv.-xx.) before meals, to which may be added nepenthe (m iii.-vi.) and acid. hydrocyan: dil. (m iii.-iv.) to relieve the pain and check the vomiting. Belladonna may sometimes be substituted for the nepenthe.

Nitrate of silver is occasionally used at St. Mary's in the treatment of gastric ulcer, with the view of relieving the pain, and of promoting the healing of the ulcer. It is important that the bowels be kept open, but all violent purgation must be avoided.

A combination of the sulphate and carbonate of magnesium (ʒss-ʒj of each) with the tincture of hyoscyamus (m xx.) will generally be found to produce the desired result. Should pain or griping result from the use of this mixture, the bowels may be kept open by means of glycerine or simple enemata. When the pain and vomiting have subsided and the patient has become convalescent, it is generally desirable to administer iron in some form. The milder preparations of iron, such as the ferri et quin. cit., or the liq. ferri dialysati are those generally used.

The following prescription is a favourite one with Dr. Broadbent in these cases: ℞ Ferri. pot. tart. gr. v, quin. sulph. gr. i-ii., acidi tartar gr. ¼, inf. calumb. ℥j. To be taken after meals. Arsenic may sometimes be added to this with advantage. The bowels must be kept regular by means of the sulphate of magnesia or soda, with which may be combined the phosphate of soda.

The diet must be carefully attended to, even after convalescence is well established. The food taken should be capable of easy digestion, and should be given at regular intervals. Change of air is attended, in most cases, with extremely beneficial results.

*Treatment of the Complications.*—It will be necessary to consider the treatment of two only of the complications of gastric ulcer, viz., hæmatemesis and perforation. The treatment of dilatation of the stomach which may occur in the course of gastric ulcer has already received separate consideration in the columns of THE HOSPITAL.

Should hæmatemesis occur, the patient must be placed in the recumbent position and kept perfectly quiet. Ice should be sucked, and an ice bag may be placed over the epigastrium. If these measures fail to stop the bleeding, half-drachm doses of the liquid extract of ergot should be given every three hours, with gallic acid (gr. x.), opium in the form of pil plumbi c. opio (gr. iii-vi.), or of liq. opii sed. (m xx.-m xxx.), given every three hours, may be also tried. Turpentine is sometimes invaluable, given as an emulsion, *i.e.*, a tea-spoonful of turpentine beaten up with the white of an egg. This may be repeated if necessary. Should everything be vomited, the subcutaneous injection of ergotine (gr. i.-iv.) or morphine (⅓ to ½ gr.), or a combination of the two must be tried. If all these measures fail, it may be remembered that hæmatemesis is rarely attended with a fatal result. Should perforation occur the recumbent position must be enforced, and all food by the mouth must be withheld. Collapse must be treated by the subcutaneous injection of ether and brandy. The brandy may be administered per rectum. Pain must be relieved by the administration of morphia. The extremities are to be kept warm by means of flannels, hot bottles, &c. The question of operation will have to be considered. The patient may be fed by means of nutrient enemata, to which brandy can be added.

